# Sex-specific association between urinary kisspeptin and pubertal development

**DOI:** 10.1530/EC-22-0165

**Published:** 2022-09-26

**Authors:** Rafaella Sales de Freitas, Thiago F A França, Sabine Pompeia

**Affiliations:** 1 Departamento de Psicobiologia, Universidade Federal de São Paulo, São Paulo, Brazil

**Keywords:** kisspeptin, metastin, urine, puberty, sex differences, adolescence

## Abstract

Kisspeptins play a crucial role during pubertal development, but little is known about how their peripheral concentrations relate to sexual maturation. This is partly due to the lack of non-invasive, quick, and reliable peripheral kisspeptin measures, which limit widespread testing. Here, we investigated the relationship between kisspeptin concentrations measured from midstream urine samples with 2-h retention periods and developmental markers (age, self-reported pubertal status, and saliva concentrations of testosterone and DHEA sulphate ) in 209 typically developing 9- to 15-year-old males and females. As a result of the study, we found marked sex differences. Kisspeptin concentrations were similar between sexes until around 12 years of age, but, thereafter, kisspeptin concentrations in females did not change significantly, whereas, in males, there was a clear positive correlation with developmental measures. Our results replicate previous findings regarding kisspeptin concentration changes across the pubertal transition obtained from blood samples, suggesting that measuring these peptides in urine has the potential for exploring kisspeptins’ peripheral effects and their associations with pubertal status.

## Introduction

Adolescence is a period of life marked by an interconnected set of social, cognitive, psycho-emotional, and morphophysiological changes (1). The morphophysiological aspects of adolescent development are related to puberty, which involves neuroendocrine changes leading to maturation of the gonads, acceleration of growth in height, and development of secondary sexual characteristics. The kisspeptin peptides, produced by small groups of neurons in the hypothalamus, play a key role in some of these pubertal changes.

In humans, kisspeptins are encoded by the *KISS1* gene, which encodes a precursor protein that can be cleaved into three shorter peptides (kisspeptins 13, 14, and 54), all of which can activate the kisspeptin receptor, KISS1R (2, 3). These peptides increase pulsatile hypothalamic gonadotropin-releasing hormone (GnRH) secretion, which in turn increases the secretion of gonadotropins by the pituitary glands, leading to the production of sex steroids by the gonads. This hormone cascade is essential for the onset and trajectory of gonadal pubertal changes and regulation of fertility (4, 5, 6).

In addition to kisspeptins’ role in the hypothalamic–pituitary–gonadal (HPG) axis, there is also evidence for the transcription of genes encoding kisspeptins and their receptor in several other peripheral tissues and organs (reviewed in (4, 7)). Together, these kisspeptin-induced central and peripheral changes are implicated not only in the regulation of reproductive processes but also in the metabolism (for recent reviews, see (8, 9, 10)) and the modulation of mood and emotion (11). Additionally, because peripheral administration of kisspeptins increases circulating gonadotropins in humans (4, 12), it seems that peripheral concentrations of these peptides can also affect the brain. This is important because puberty is a complex, multisystemic process that is influenced by several factors, including genetic, metabolic/nutritional, socioemotional factors, and socioeconomic status (SES) (1). This suggests that kisspeptins may play an integrative role in pubertal development so quantifying peripheral, circulating kisspeptin concentrations holds promise to improve our understanding of puberty and the characterization of the pubertal trajectory. However, there is still a considerable amount of controversy in the literature regarding changes in circulating kisspeptins during puberty in humans.

For instance, some studies have shown that, in girls, kisspeptin concentrations measured in blood increase from pre-puberty to the initial stages of puberty but not from then on (13, 14, 15). Girls with idiopathic central precocious puberty also present higher serum concentrations of kisspeptins than normally developing controls (16, 17), further suggesting a role of peripheral kisspeptins in initiating puberty in females. However, Zhu* et al*. (15) found a marked increase in serum concentrations from the first stages of puberty in boys, but not in girls, in whom concentrations were much lower and more stable after pubertal onset. The picture is further complicated by results from Pita* et al*. (18), who did not find changes in plasma kisspeptins from prepubertal to pubertal youngsters of either sex. Some of the inconsistencies may be related to the sexual dimorphism in kisspeptin expression, its serum concentrations, and the distribution of kisspeptin receptors, which are believed to explain some of the developmental differences between males and females (19, 20). However, this does not address all the above-mentioned inconsistencies.

Additional questions about the role of kisspeptins in puberty arise when we consider that the activation of the HPG axis is not the only change that occurs during puberty (21). Apart from gonadal changes that result from this activation, which include the increased production of hormones such as testosterone and oestrogens, changes in genitalia in both sexes, breast growth in girls and facial hair growth, and voice changes in boys, puberty also involves alterations in the adrenal glands, independently but, to a certain extent, is parallel to maturation of the gonadal axis (21, 22). These changes lead to increases in adrenal androgen concentrations and contribute to the development of other secondary sexual characteristics, such as growth of body and pubic hair and skin changes (21, 22). Accordingly, there seems to be a positive linear correlation between the concentrations of peripheral kisspeptins and adrenal androgens such as DHEA and its main metabolite, DHEA sulphate (DHEA-S) (15, 23). As this brief overview of the literature shows, the role of peripheral kisspeptin concentrations and pubertal development still needs much clarification.

In humans, kisspeptin concentrations can be consistently quantified not only in blood samples (24) but also in urine (25). Urine kisspeptin detection is possible because these peptides consist of small molecules that pass through the glomerular filtration membrane in the kidneys (26). However, while serum and urine measures of kisspeptins present the same overall pattern of variation, measurements made in urine samples are less sensitive (25). Notwithstanding, urine detection of kisspeptins has advantages to compensate for this limitation. Urine collection is a non-invasive method, a highly desirable characteristic when working with vulnerable populations, such as children and adolescents. Moreover, urine samples are easier and cheaper to collect than blood samples, the latter requiring trained staff to draw blood.

While previous studies (25, 26) showed that measuring kisspeptins from urine samples can be a good, non-invasive method for measuring these peptides, they did not provide detailed information about sample collections and analyses. For example, these reports do not specify factors such as urine retention time, if midstream urine was used or if urine was collected just once or over a period of many hours. Also, urine assays used in these studies were carried out in pregnant women (25) and women of reproductive age over the menstrual cycle (26), so it is unknown if this would be a sensitive method to detect changes in peripheral kisspeptin concentrations during pubertal development.

In this context, our objective in this study was to test whether urine detection of kisspeptins in typically developing early male and female adolescents relates to classic developmental markers such as chronological age, as well as a set of indicators of pubertal status, including measures based on physical changes (scores on the Pubertal Developmental Scale) and hormone concentrations (testosterone as a biomarker related to gonadal pubertal changes and DHEA-S, a marker of adrenal sexual maturation). Due to lack of information on urine samples used in prior studies that assessed urine kisspeptins, we collected midstream urine with a minimum of 2-h retention to establish if this is a feasible method to measure changes in kisspeptin concentrations related to pubertal development.

## Methods

### Participants

Our cross-sectional study involved a convenience sample composed of 209 (120 females) healthy, native Portuguese speaking, 9- to 15-year-old youngsters – an age interval spanning the full range of pubertal development stages in most adolescents (21). Participants were only included after obtaining signed informed consent from their guardians and their own assent. Exclusion criteria were: (i) use of chronic medication (chosen as a criterion to rule out the presence of clinical disorders), (ii) use of drugs that could impact pubertal development and metabolism, and (iii) having been held back in school for a year or more and/or being a student with special needs (both of which could indicate the presence of clinical and/or cognitive issues).

### Procedure

The study protocol was approved by the Committee of Ethics in Research of the Federal University of São Paulo (# 56284216.7.0000.5505). All participants and their guardians provided informed assent and consent, respectively, as per local ethical guidelines. Participants were recruited from schools in the city of São Paulo, Brazil. The guardians provided demographic and health information. Data from participants were obtained at their own school and included anthropometric measurements, self-rated pubertal status, and saliva and urine samples, which were collected between 10:00 and 16:00 h. Additional information about the methods and measures can be found in the Supplementary Methods file (https://osf.io/dqesj/).

### Measures

#### Urinary kisspeptin concentrations

Midstream urine samples of approximately 10 mL were obtained after at least 2-h retention periods and were kept in ice-cooled Styrofoam boxes for up to 5 h during transport until they were centrifuged at 1000 ***g*** for 20 min, after which the supernatant was aliquoted at 1200 μL. Aliquots were stored in a freezer at −80°C until the analyses. Concentrations of kisspeptins were determined by enzyme-linked immunosorbent assays (ELISA) for human samples following the supplier's guidelines, in uniplicate (KIT Cloud-Clone Corp. (CCC, Wuhan, China)). The kit used antibodies produced against the full human KISS1 protein, minus the signal peptide. In other words, it used the sequence from Glu20 to Gly138. This sequence encompasses kisspeptins 54 and its shorter forms. Detection range is 31.2–2000 pg/mL, with minimum detectable dose of 13.1 pg/mL. Intra- and inter-assay coefficients of variance are <10% and <12%, respectively.

#### Other developmental markers

Participants’ age was considered in number of months. To assess pubertal status, we used the self-reported Pubertal Developmental Scale (PDS), adapted from Carskadon and Acebo (27) for local use by Pompeia* et al*. (28). The scale contains a series of questions regarding the development of secondary sexual characteristics related to both gonadal and adrenal factors: body hair development, growth spurt and skin changes for participants of both sexes, voice changes and facial hair growth for boys, and breast growth in girls. All these were measured using a 4-point Likert scale (ranging from 1 = ‘not yet started’ to 4 = ‘seems complete’; with 0 = ‘I don't know’, coded as missing data). Girls were also asked to indicate whether they already had their first menstruation (menarche, score as 1 = no, or 4 = yes). The final scores were the mean ratings in all five sex-specific questions (27). We also assessed pubertal development using two hormonal measures obtained from saliva: free salivary testosterone (a marker of gonadal development) and DHEA-S concentrations (a marker of adrenal changes). These markers were chosen because their concentrations reportedly increase throughout the pubertal trajectory in both sexes, whereas, others, like oestrogens, only consistently increase in girls (29). Saliva samples were free of visual blood traces and were collected by passive drool 10 min after a light mouthwash with water. Testosterone and DHEA-S concentrations were determined from saliva aliquots (700 μL) using ELISA kits (Salimetrics, State College, PA, USA) following the supplier’s guidelines. The test sensitivities were 0.94 pg/mL and 0.05 ng/mL, for testosterone and DHEA-S, respectively. Samples were assessed in uniplicate, but some random samples were tested in duplicates. In these, the mean coefficients of variation were 4.4% for testosterone samples and 4.1% for DHEA-S.

#### Other control measures

Because socioeconomic and metabolic status can affect pubertal status and kisspeptin concentrations (1, 7, 9, 15, 18, 19), we controlled for these factors. Participants’ SES was assessed based on family purchasing power, in accordance with the guidelines of the Brazilian Association of Market Research (http://www.abep.org.br; English version http://www.abep.org/Servicos/Download.aspx?id=11). This questionnaire, answered by one of the guardians, attributes points based on the family’s access to objects and services of value (e.g. car, freezer, housemaid), parents’ educational level, and neighbourhood characteristics.

BMI (kg/m^2^) was used as a proxy for metabolic status and was calculated using weight, determined after removal of coats and shoes with an OMRON HBF-514C Body Control scale (to the nearest 0.1 kg) and standing height (to the nearest cm), recorded in bare feet using a stadiometer. Both weight and height were measured twice, and the average values were used.

#### Statistical analyses

The descriptive and inferential analyses were performed with the SPSS, v. 21. Pearson correlation indices were used to explore the relationship between kisspeptin concentrations and markers of development (age, PDS, testosterone, and DHEA-S). Using univariate General Linear Models (GLM), we investigated how the urinary concentrations of kisspeptins (treated as a dependent variable) related to developmental indicators (each in a separate model) by sex (modelled to interact with the developmental markers), adjusting for SES and BMI. Multiple R^2^ values of the whole models ranging from 0.13–0.25 were considered medium effect sizes and large effect sizes when above 0.26 (30). For each variable, effect sizes were assessed with the partial eta-squared statistic (0.0588–0.1378 = medium; >0.1379 = large) (31). The level of significance adopted for all the inferential analyses was *P* ≤ 0.05. There was neither imputation of missing data nor removal of outliers.

## Results

The sample represented participants in all different stages of sexual maturation, varying ages, SES, and BMI (Table 1). These variables were all regressed on the self-assessed PDS scores, and the resultant intercepts and unstandardized regression coefficients (B, with their respective 95% CI) are also reported in Table 1 so that the reader can gauge the values per sexual maturation status (PDS scores). There were some missing data (four (one for girls and three for boys) for DHEA-S, three (one for girls and two for boys) for testosterone, and four (two for girls and two for boys) for BMI). The databank can be found at https://osf.io/dqesj/.

**Table 1:** Descriptive data (mean, standard deviation (s.d.), minimum (Min), maximum (Max)) of all analysed variables included in this study, by sex, and intercept (Interc.) and unstandardized regression coefficients (B) (with respective 95% CI) for the regression of each variable on scores in the self-assessed Pubertal Development Scale (PDS).

Variable	Girls (*n* = 120)										Boys (*n* = 89)									
	Mean	s.d.	Min	Max	Interc.	±95% CI		B	95% CI		Mean	s.d.	Min	Max	Interc.	±95% CI		B	B ±95% CI	
PDS (score)	2.7	0.7	1.2	3.8							2.1	0.6	1.0	3.6						
Age (years)	12.6	1.7	9.0	16.0	8.03	7.20	8.86	1.69	1.41	1.98	12.2	1.7	9.0	15.0	7.99	7.11	8.87	2.00	1.62	2.39
Kisspeptin (pg/mL)	47.29	7.72	32.93	72.03	48.131	42.357	53.905	−0.311	−2.381	1.758	78.69	34.77	32.79	211.08	18.733	−3.130	40.596	28.401	18.482	38.320
Testoterone (pg/mL)	2.66	1.85	0.36	11.58	11.126	−2.256	24.509	6.497	1.779	11.214	2.89	1.96	0.12	11.28	−48.494	−77.592	−19.396	48.289	35.041	61.536
DHEA-S (ng/mL)	29.77	20.17	4.18	112.83	0.709	−0.509	1.928	0.694	0.265	1.124	50.93	46.87	3.13	254.12	0.905	−0.461	2.271	0.965	0.339	1.591
BMI (kg/m^2^)	20.9	3.8	14.5	31.2	16.34	13.57	19.10	1.78	0.80	2.75	21.1	4.2	13.7	32.8	17.86	14.93	20.79	1.48	0.16	2.80
SES (score)	7.46	2.82	2.00	15.67	6.62	4.52	8.72	0.312	−0.44	1.07	8.04	3.12	2.38	19.67	5.10	2.89	7.30	1.40	0.40	2.40

B, unstandardized regression coefficient (reflect the change in the outcome variable for every 1-unit of change in the PDS score); DHEA-S, DHEA sulphate ; SES, family socioeconomic status *per capita.*

Pearson correlations of kisspeptin concentrations with the developmental markers were all positive and statistically significant (*P*-values <0.01) for boys: for age, r = 0.624; for PDS, r = 0.595; for DHEA-S, r = 0.277; for testosterone, r = 0.350. None of the correlations were significant for girls (coefficient values ranged from r = −0.027 to .132).

**Figure 1:**
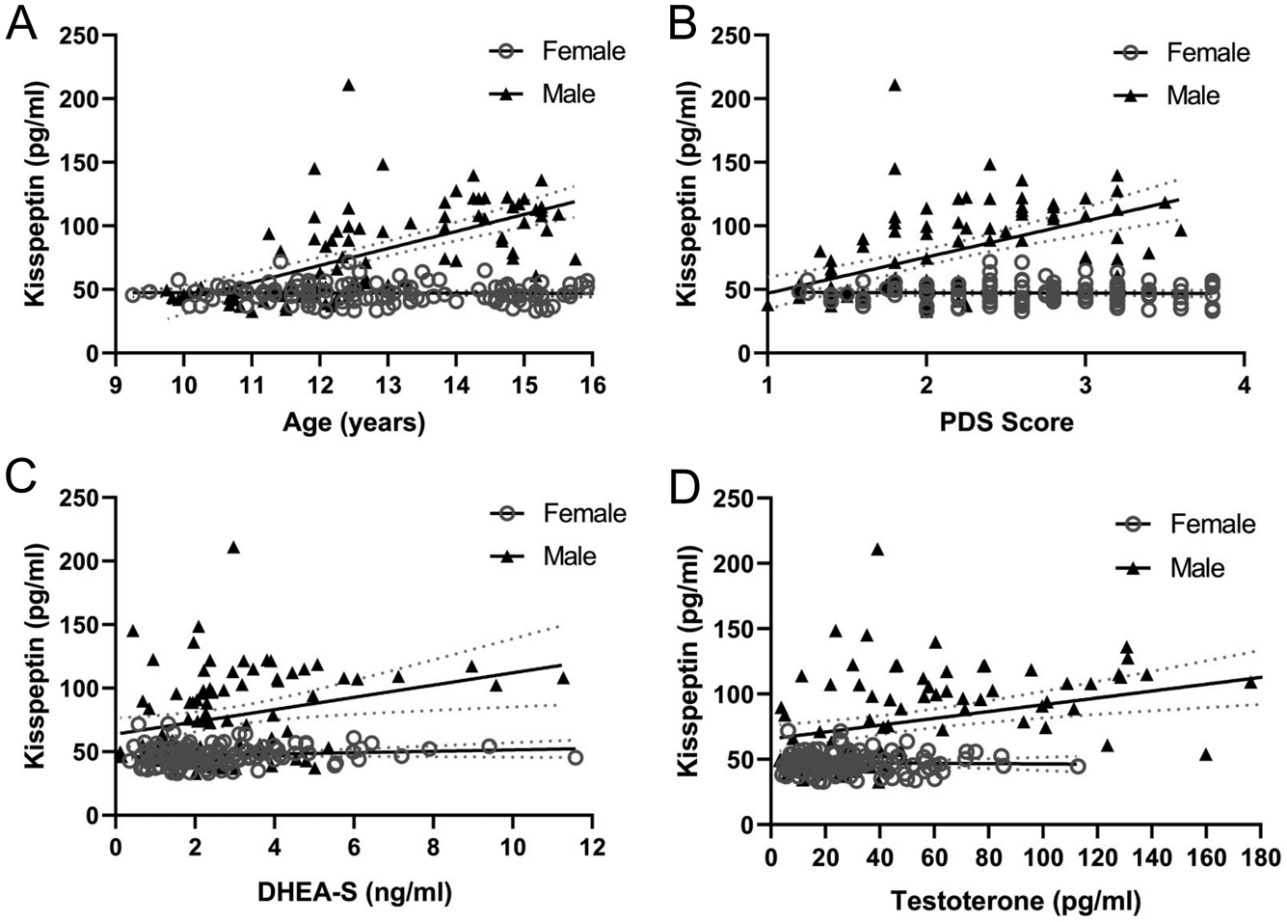
Scatterplots representing individual data of urine kisspeptin concentrations per sex according to developmental markers (A, age in months; B, PDS score; C, DHEA-S; D, testosterone). Continuous lines represent simple regressions, and dotted lines represent their 95% CIs. PDS, scores in the Pubertal Development Scale; DHEA-S, DHEA sulphate. Kisspeptin concentrations were positively correlated and statistically significant (*P*-values <0.01) with all developmental markers for males: for age, r = 0.624; for PDS, r = 0.595; for DHEA-S, r = 0.277; for testosterone, r = 0.350. No statistically significant correlations were found for females (coefficient values ranged from r = −0.027 to 0.132; *P*-values <0.05). This representation is not corrected for socioeconomic status and BMI, which were non-significant predictors (see main text).

Figure 1 shows kisspeptin concentrations according to each developmental marker, separately by sex. The plot in Fig. 1A shows the relationship between kisspeptin concentrations and age in boys and girls. We can see that there is no longer overlap of the 95% CIs between kisspeptin levels of girls and boys from just before the age of 12 years onward, with boys showing higher concentrations thenceforth, while girls show no perceptible variation in kisspeptin concentrations at any age. Regarding PDS scores (Fig. 1B), boys had higher kisspeptin concentrations than girls from PDS score 2 onward. For DHEA-S and testosterone (Fig. 1C and D plots, respectively), sex differences are observed in the full range of results, again with boys presenting higher kisspeptin concentrations. In all cases, kisspeptin concentrations in girls were unchanged with regards to developmental measures.

Table 2 shows the results of the GLMs used to investigate how kisspeptin concentrations (dependent variable) varied according to developmental variables and sex in separate models (age in months, PDS scores, DHEA-S, and testosterone). All models were able to explain a significant proportion (>34%) of the variance in urine kisspeptin concentrations. All developmental markers had significant effects (*P* values <0.002), except for testosterone, which fell short of significance (*P*  = 0.051). Kisspeptins increased with age (B = 1.13 pg/mL per month), PDS scores (B = 28.96 pg/mL per point), DHEA-S (B = 4.78 pg/mL per ng/mL), and testosterone (B = 0.25 pg/mL per pg/mL), but effect sizes in terms of partial eta-squared values only reached medium values in the first two cases. Sex was a significant predictor in all models (*P* values <0.006), with boys having consistently higher kisspeptin concentrations than girls. The interaction of sex with each developmental marker was also significant (*P* values <0.02), indicating that kisspeptin concentrations were not similar among boys and girls throughout the tested ages. However, this interaction reached medium effect sizes only for the models with age and PDS scores. BMI and SES were not significant factors in any of the models.

**Table 2:** Results of the general linear models (GLM) used to determine urine kisspeptin concentrations across development (chronological age and pubertal indicators), per sex, controlled for BMI and socioeconomic status.

Models (df)	Predictors	F	*P*	pη^2^	B	Multiple R^2^ (adjusted R^2^)
1 (1204)	Age (months)	64.61	<0.001	0.245	1.126	
	Sex (males as a reference)	44.32	<0.001	0.182	140.437	
	Interaction sex vs age	68.68	<0.001	0.257	−1.125	0.56 (0.55)
	BMI	0.83	0.36	0.004	−0.319	
	Socioeconomic status	0.21	0.65	0.001	0.214	
2 (1204)	PDS (score)	38.945	0.001	0.164	28.956	
	Sex (males as a reference)	7.71	0.006	0.037	30.329	
	Interaction sex vs PDS	42.70	<0.000	0.177	−29.200	0.49 (0.48)
	BMI	0.29	0.59	0.001	−0.203	
	Socioeconomic status	0.04	0.84	<0.001	0.104	
3 (1200)	DHEA-S (ng/mL)	10.09	0.002	0.049	4.782	
	Sex (males as a reference)	10.50	0.001	0.051	−8.391	
	Interaction sex vs DHEA-S	5.86	0.02	0.029	−4.096	0.36 (0.34)
	BMI	0.28	0.59	0.001	−0.220	
	Socioeconomic status	1.09	0.30	0.006	0.583	
4 (1201)	Testosterone (pg/mL)	3.84	0.05	0.019	0.252	
	Sex (males as a reference)	12.55	0.004	0.600	−18.292	
	Interaction sex vs testosterone	5.54	0.02	0.029	−0.274	0.39 (0.38)
	BMI	0.05	0.83	<0.001	0.089	
	Socioeconomic status	0.36	0.55	0.002	0.337	

Adjusted R^2^, R^2^ adjusted for the number of predictors in the model; B, unstandardized regression coefficient (all other variables held constant, it reflects the degree of change in kisspeptin concentrations for every 1-unit of change in the predictor); DHEA-S, DHEA sulphate; F-value, F-statistic (i.e. the ratio of the between- and within-group variances of the group means divided by the mean of the within-group variances); multiple R^2,^ the variance in kisspeptin concentrations explained by the model; partial eta-squared (pη^2^), the proportion of variance explained by each variable of the total variance remaining after accounting for variance explained by the other variables in the model; PDS, scores in the Pubertal Development Scale.

## Discussion

In this exploratory study, we aimed to show the feasibility of measuring kisspeptin concentrations from midstream, 2-h urine retention samples as a method to quantify this peptide according to developmental markers (chronological age and pubertal markers based on physical maturation and hormonal concentrations) in the first half of adolescence. The results showed that kisspeptin concentrations were very similar in boys and girls in early puberty (PDS scores <2), around the ages of 9–11 years, but that, thenceforth, these concentrations markedly increased in boys but did not change in girls. These sex differences corroborate the data of Zhu* et al*. (15), who measured kisspeptins in the serum of typically developing youngsters. Furthermore, the fact that the study by Zhu* et al*. (15) had, like ours, a larger sample size compared to other published studies that measured kisspeptin in blood samples, and used the same commercial kit employed in our study, suggests that urine measurement is a feasible, non-invasive method to quantify peripheral kisspeptins that can be used as an alternative to measuring these peptides in blood samples. However, irrespective of the type of sample (urine or blood), further studies are required to confirm the reliability of the kit, as discussed below.

The only difference between Zhu* et al*.’s (15) and our study was that they found that serum kisspeptin concentrations in girls were higher at the beginning of puberty compared with pre-puberty, confirming the findings of Xiaoyu* et al*. (14). This change in kisspeptin levels at the onset of puberty is also consistent with the findings of Yang* et al*. (16), who showed that girls with idiopathic central precocious puberty had higher serum levels of kisspeptins than normally developing controls and that, after treatment, these concentrations fell below pre-treatment levels, suggesting a role of peripheral kisspeptin concentrations on pubertal onset in this sex. We did not detect this specific increase in the present study, probably due to the small number of prepubertal girls in our sample – only 17 girls scored less than 2 in the PDS, compared to 36 boys. Furthermore, Zhu* et al*. (15) used a discrete marker of pubertal status (Tanner stages), while we used a continuous PDS score that makes this putative effect difficult to show.

Of note, the data from Zhu* et al*. (15), Xiaouy* et al*. (14), Yang* et al*. (16), and ours are neither in line with the results of Pita* et al*. (17), who found stable serum kisspeptin concentrations throughout puberty and adulthood nor findings of Jayasena* et al*. (13), who showed that plasma concentrations of kisspeptin peaked between the ages of 9 and 12 years in both sexes, after which they decreased until adulthood. It is difficult to pin down the roots of the disagreement between published studies. On the one hand, there are differences between studies in the methods used to assess kisspeptins. Pita* et al*. (18) and Jayasena* et al*. (13) quantified kisspeptins using radioimmunoassays with antibodies produced against kisspeptin 54 that were capable of detecting kisspeptins 14 and 13/10 and had no cross-reactivity with other human RF-amide peptides bearing structural similarity to kisspeptin. Our study and the ones from Zhu* et al*. (15), Xiaoyu* et al*. (14), and Yang* et al*. (16) all used ELISA kits. The kit used by Yang* et al*. (16) employed antibodies produced against human kisspeptin 54, but the manufacturer states they cross-reacted with some peptides from the RF-amide family. There is no information about the kit used by Xiaoyu* et al*. (14), while the kit employed by Zhu* et al*. (15) and by the present study used antibodies produced against the product of the KISS1 gene, which includes the kisspeptin 54 sequence but also several additional amino acids. This manufacturer (KIT Cloud-Clone Corp. [CCC, Wuhan]) states the kit has high sensitivity and specificity, but there is limited information about cross-reactivity with other RF-amide peptides and about specificity for particular kisspeptins. How the differences in antibodies and detection methods used in the different studies affect the results is unknown and requires further investigation, especially because this variability in kisspeptin measurement methods is ubiquitous in the literature.

On the other hand, while it is tempting to give more weight to the results of Pita* et al*. (18) and Jayasena* et al*. (13) on the grounds of the apparently lower risk of bias in their kisspeptin measurement methods, it is important to note that this is not the only difference between the studies. For example, in contrast to the relatively large sample size used in the present study and in Zhu* et al*. (15), both Pita* et al*. (18) and Jayasena* et al*. (13) used very small samples – especially considering the analyses by sex and age during puberty. This, and the fact that those studies apparently did not control for multiple comparisons, increases the risk of unreliable results.

As shown here, peripheral kisspeptin concentrations have been found to be positively associated with testosterone and DHEA-S in pubertal boys (15). However, we failed to find reports of these correlations in pubertal girls. Importantly, the fact that DHEA-S is related to kisspeptins does not necessarily mean that these peptides act on adrenal pubertal systems. Such an association would be expected due to the relative synchronicity between gonadal and adrenal maturation, despite the independent nature of these processes (21). Although higher concentration of testosterone and DHEA are found in more advanced pubertal stages in both sexes (29), Van Hulle* et al*. (32) claim that these hormones neither vary in parallel with changes in secondary sexual characteristics measured by subjective scales nor seem to share the same mechanisms in males and females (32). This can help explain why these hormones were only related to kisspeptins in boys, although they only reached small effect sizes.

Overall, our results, together with those of studies in other pubertal samples, show that increases in peripheral kisspeptins are not only associated with the initiation of puberty but are also related to sexual maturation trajectory in boys, while girls present much lower concentrations of peripheral kisspeptins and show little developmental variation except from the passage from pre-puberty to early puberty (14, 15). These sex differences may seem puzzling at first, but some additional lines of evidence regarding the roles of kisspeptins in development and beyond can shed some light on the meaning of these results. First, there are sex differences in the sensitivity and/or number of central KISS1 receptors (19, 20, 33) that can also vary across puberty, at least in boys (33). Secondly, kisspeptins are also produced by several peripheral tissues that are sex-specific, including the testes and ovaries, and may have sex-specific roles in the reproductive systems of males and females that go beyond those related to puberty itself (as reviewed in (8)). Thirdly, due to the widespread expression of kisspeptin in the periphery beyond the gonads, including in the adrenal glands, liver, pancreas, adipose tissue, and blood vessels (reviewed in (7)), at least part of the sex differences in peripheral kisspeptin concentrations may well be explained by other physiological roles of kisspeptins. In particular, emerging evidence, mainly from animal and* in vitro* studies, suggests a role for kisspeptins in metabolism regulation (for reviews, see (7, 9, 34)). While the metabolic effects of kisspeptins seem to differ between sexes (35), overall, the evidence from their involvement in glucose and lipid metabolism suggests that higher levels of kisspeptins are related to higher energy expenditure (7, 9, 34). Notably, the higher concentrations of kisspeptins in pubertal boys observed in our study seem to be in line with known sex differences in metabolism and energy expenditure and are known to be lower in females (reviewed in (36)).

We conclude that determining (midstream) urinary kisspeptin concentrations with a 2-h retention period may be a feasible and sensitive method to quantify circulating levels of these peptides in pubertal populations. The sex differences that were found confirmed results from serum assays in typically developing early adolescents and involved increases in concentrations from early puberty in boys, in contrast with lower and more stable concentrations in girls (except for the transition from pre- to early puberty). Non-invasive methods such as this may aid in the understanding of the differential role of kisspeptins in male and female puberty, such as sex differences in metabolism (7, 9, 34) and psychiatric disorders (1), especially considering that kisspeptins seem to modulate various systems that affect behaviors such as mood, fear, and anxiety (11).

Peripheral urinary measurement of kisspeptins can also be helpful in the diagnosis of central precocious puberty, since the concentration of kisspeptins is higher in affected individuals compared to typically developing same-age peers (19, 37). This condition is much more common in girls (19) and is possibly related to the effects of kisspeptins in triggering puberty in this sex. In contrast, there is a much higher incidence of delayed puberty in boys (19), which might be associated with malfunctioning of the mechanisms that lead to the sharp increase of kisspeptins in peripheral tissues as puberty progresses. A comprehensive understanding of the expression, function, and potential molecular mechanisms of kisspeptin/KISS1R in the peripheral reproductive system can also contribute to our general knowledge of typical sexually dimorphic patterns of pubertal development. However, progress in this area will also depend on studies trying to disentangle the reproductive/developmental effects of kisspeptins from their other physiological roles, such as regulation of metabolism, and on investigations of the possible crosstalk between these functions, so that we can gain a deeper understanding of the biological meaning of variations in peripheral kisspeptin concentrations.

Regarding limitations, the cyclicity of the HPG axis pulses may influence variations in kisspeptins in females (25), which were not controlled here. This was also not controlled in any of the prior studies with pubertal samples, so this does not invalidate the comparisons that were made with other studies. The same can be said about the lack of perfect control over the period of the day in which saliva and urine samples were collected, which ranged from 10:00 to 16:00 h. Had we included more prepubertal girls, or used Tanner staging, it is possible that we would have shown the increase in kisspeptins from this stage to early puberty that was reported by other researchers (15). On the upside, unlike most of the published literature, we tested a sample from a developing country, with wide variations in SES and BMI, both of which were adjusted for.

## Declaration of interest

The authors declare that there is no conflict of interest that could be perceived as prejudicing the impartiality of the research reported.

## Funding

This work was supported by the São Paulo Research Foundation (FAPESP: # 2016/14750-0, 2018/06374-3, and 2019/11706-8), as well as CAPES (finance code 001), AFIP, and CNPq (#301899/2019-3).
